# Editorial: Translational research in neuropathic pain: Current status and future directions

**DOI:** 10.3389/fpain.2022.1024013

**Published:** 2022-09-09

**Authors:** Jay Karri, Ameet S. Nagpal, Sheng Li

**Affiliations:** ^1^ Department of Orthopedic Surgery and Department of Anesthesiology, University of Maryland School of Medicine, Baltimore, MD, United States; ^2^ Department of Orthopedics and Physical Medicine, Medical University of South Carolina, Charleston, SC, United States; ^3^ Department of Physical Medicine and Rehabilitation, McGovern Medical School, University of Texas HSC at Houston, Houston, TX, United States

**Keywords:** neuropathic pain, future directions, preclinical (*in vivo*) studies, translational research, clinical research

**Editorial on the Research Topic** Translational research in neuropathic pain: Current status and future directions by Karri J, Nagpal A and Li S. (2022) Front. Pain Res. 3: 1024013. doi: 10.3389/fpain.2022.1024013

The pathophysiology of neuropathic pain and the neuromodulation of underlying mechanisms by various interventions (pharmacological and non-pharmacological) has yet to be clearly elucidated (1–3). Consequently, neuropathic pain can be clinically challenging, and many patients are refractory to standard of care treatments (4, 5). Although preclinical studies exploring neuropathic pain mechanisms and interventions have been robust in recent years, advances in clinical care have been relatively lacking (6–8). This disparity is largely thought to be a result of limited, well-designed translational pain studies. This editorial aims to highlight the current state of translational research, new directions in animal models, objective assessments and measures of pain, and personalized medicine, to better our understanding of challenges in translating pre-clinical research to human patients.

## State of current research and new directions

### Approaches in translational research

While translational research has been long simplified as the implementation of basic science findings into the clinical setting, it has been increasingly recognized that such linear and unidirectional approaches may be limited given the vast differences in pain physiology, perception, and modulation that exist between animal models and human patients (6–8). Rather, with growing appreciation for the multidimensional aspects of chronic pain, it has been increasingly recognized that translational research needs to be bidirectional. Such bidirectional efforts will serve to prove more fruitful in curating basic science and clinical exploration with accommodations for bench to bedside differences of the multidimensional pain experience.

### Objective measures and assessments

The need for objective and reproducible pain assessments has been a long-heralded goal for scientists and clinicians alike (6, 7). As aforementioned, the multidimensional components of pain make it challenging to clearly distinguish sensory, perception, and emotional components of pain between mouse and man. Despite these limitations, there has been an ever-increasing utilization of novel pre-clinical pain models (9). Expectedly, these increases in models and depth of research have not translated into meaningful clinical advances (9–11). Given the clear need for objective pain measures, there has been some advancement in neuroimaging any sympathetic testing as surrogate measures for chronic pain (12–19). Neuroimaging methods, including fMRI and PET studies, have revealed some promising insights into anatomical and physiological aspects of chronic pain, both at baseline and in response to noxious and analgesic stimuli (12–14). Regarding sympathetic testing, quantitative sudomotor axon reflex testing, skin conductance measures, and heart rate variability testing have shown promise in objectively producing parameters of pain and analgesic response (15–19). However, such assessments are limited as they represent surrogates of the sensory-perceptive components of pain, without measuring or distinguishing the affective-motivational aspects.

### Personalized medicine and focus on implementation

With the era of personalized medicine, there has been an emerging interest for delivering patient-specific, targeted treatments (9). Patient stratification into various neuropathic pain phenotypes, largely by genetic and/or genomic analyses, may potentially allow for profiling of implicated nociceptors, metabolization capacities, and provide opportunities for targeted treatment. While seemingly promising, Dib-Hajj et al. and others report that extreme variations in animal models and biomarkers have hampered such advances, which are currently far from current clinical standard (9). Consequently, others have rightfully advocated for advancement by optimizing implementation of already evidenced treatments. Such efforts in implementation have largely surrounded understanding and addressing differences in pharmacodynamics and drug distribution to enhance medication dosing. Notably, such mismatches also explain gaps in translating findings from animal studies to human patients.

## Articles published in the research topic

The scope of this editorial serves to frame the context of these studies and allows readers to recognize both their potential when applied to humans, and possible barriers in translating findings to the clinical space.

Karri et al (2022): Recent advances from SCI-specific animal studies and translational models have furthered our understanding of neuronal excitability, glial dysregulation, and chronic inflammation processes that mediate neuropathic pain. These developments have subsequently facilitated exploration of spinal neuromodulation as a treatment modality. In this narrative review, we provide a comprehensive review of the pathogenesis of neuropathic pain following SCI, intrathecal drug delivery as a treatment, and novel pharmacologics across impactful animal and clinical studies.

Kwilasz et al (2022): Previously, it has been shown that toll-like receptor 4 (TLR4) antagonists, such as (+)-naltrexone [(+)-NTX], block neuropathic pain and associated spinal inflammation in rats. In this study, Kwilasz et al. utilize a novel rat model of EAE to extend these findings to demonstrate that (+)-NTX blocks TLR2 in addition to TLR4, as well as completely prevented the EAE-induced memory impairments and robustly attenuated the associated proinflammatory effects.

Shillo et al (2021): Via robust immunobiological studies of gathered skin biopsies from patients with type 2 diabetes and healthy volunteers, Shillo et al. found that diabetics with painful neuropathy had increased dermal vasculature and decreased sub-epidermal nerve fiber density relative to diabetics with painless neuropathy. They thus conclude that this hypoxia-induced increase of vasculature may expose associated nociceptor fibers to a relative excess of algogens, thus leading to painful-DPN.

Andressa de Souza et al (2021): Neuromodulation by tDCS as an adjunct therapy operates by facilitating cortical plasticity to enhance the effectiveness of therapies. It's being a non-invasive intervention also makes it an attractive option for treating various pain syndromes, including post-stroke shoulder pain as studied here. However, the authors demonstrated that physical therapy combined with active tDCS did not yield any additive analgesic benefits compared to physical therapy alone, likely due to a ceiling effect of the physical therapy.

Nagpal et al (2021): The abuse liability associated with opioid medications may possibly be diminished with use of adjunct atypical antipsychotics, which could block dopamine reward associated with opioids. In this pilot, Nagpal et al. found that the antipsychotic-opioid combination did not alter analgesic responses to thermal stimuli or increase the incidence of adverse effects. Such findings are critical for future development of analgesic drug combinations and facilitate future study of abuse potential in drug users.

Joksimovic et al (2022): In this review, the current mechanistic understandings of painful diabetic neuropathy are summarized, with a focus on nociceptive ion channels. These nociceptive ion channels are of particular interest as novel pharmacologic targets given long established successes in neuromodulating these targets in animal models.
